# New advances in mechanomyography sensor technology and signal processing: Validity and intrarater reliability of recordings from muscle

**DOI:** 10.1177/2055668320916116

**Published:** 2020-04-09

**Authors:** Claire Meagher, Enrico Franco, Ruth Turk, Samuel Wilson, Nathan Steadman, Lauren McNicholas, Ravi Vaidyanathan, Jane Burridge, Maria Stokes

**Affiliations:** 1 School of Health Sciences, University of Southampton, Southampton, UK; 2 Department of Mechanical Engineering, Imperial College London, London, UK; 3 Centre for Sport, Exercise and Osteoarthritis Versus Arthritis, Nottingham, UK

**Keywords:** Rehabilitation, sensors/sensor applications, rehabilitation devices, upper-limb, electromyography, sensor design, mechanomyography

## Abstract

**Introduction:**

The Mechanical Muscle Activity with Real-time Kinematics project aims to develop a device incorporating wearable sensors for arm rehabilitation following stroke. These will record kinematic activity using inertial measurement units and mechanical muscle activity. The gold standard for measuring muscle activity is electromyography; however, mechanomyography offers an appropriate alterative for our home-based rehabilitation device. We have patent filed a new laboratory-tested device that combines an inertial measurement unit with mechanomyography. We report on the validity and reliability of the mechanomyography against electromyography sensors.

**Methods:**

In 18 healthy adults (27–82 years), mechanomyography and electromyography recordings were taken from the forearm flexor and extensor muscles during voluntary contractions. Isometric contractions were performed at different percentages of maximal force to examine the validity of mechanomyography. Root-mean-square of mechanomyography and electromyography was measured during 1 s epocs of isometric flexion and extension. Dynamic contractions were recorded during a tracking task on two days, one week apart, to examine reliability of muscle onset timing.

**Results:**

Reliability of mechanomyography onset was high (intraclass correlation coefficient = 0.78) and was comparable with electromyography (intraclass correlation coefficient = 0.79). The correlation between force and mechanomyography was high (R^2^ = 0.94).

**Conclusion:**

The mechanomyography device records valid and reliable signals of mechanical muscle activity on different days.

## Introduction

The ability to measure muscle activity to aid recovery in the home environment may enhance self-management in neurological rehabilitation. An interactive system, Mechanical Muscle Activity with Real-time Kinematics (M-MARK), is being developed to aid recovery of function after stroke.^1^ The M-MARK system is a home-based class-one medical device for stroke upper-limb rehabilitation. The system incorporates wearable sensors for arm rehabilitation at home. These will record kinematic activity using inertial measurement units (IMUs) and mechanical muscle activity using mechanomyography (MMG) to assess the quality of movement of the stroke affected upper-limb as individuals perform arm tasks related to activities of daily living.

The IMU sensors are connected by poppers to a light, breathable garment developed with extensive end user testing. The position of the MMG sensors is initially determined by a therapist performing a clinical assessment of the muscle body location and choosing from an array of pre-defined holes within the garment. This is to ensure that the MMG is placed accurately on the relevant muscle body for each individual. Once the position is determined by the therapist, a simple clip is placed into the relevant holes and remains there for subsequent use. The person with stroke can then easily attach and detach the MMG sensors via a simple pull string and clip-in mechanism. The system was specifically designed together with people who have had a stroke. This ensured a high level of usability, which is essential for a home-based rehabilitation system.

Electromyography (EMG) for recording electrical muscle activity has been available for many years but has several limitations for use outside the clinical environment.^2^ MMG is an alternative to EMG that measures muscle vibrations (i.e. mechanical activity) using a sensor, such as a microphone or accelerometer.^3^ Validity of MMG signals, in terms of recording known vibration frequencies, has been determined.^4^ Before MMG can be used in the M-MARK system, its validity and reliability of recording signals from muscles needs to be established.

The MMG field has a colourful history, which began in 1665 when Francesco Maria Grimaldi, a Jesuit priest and scientist, discovered that muscles make rumbling sounds.^3^ The field remains largely unrecognised and has been regularly rediscovered throughout the centuries.^5^ Much of the pioneering work on developing the MMG technique (formerly termed acoustic myography) was conducted by Dr Dan Barry,^6^ who was the first to demonstrate that MMG signals are generated by lateral oscillations of muscle fibres. He also investigated clinical applications of MMG, including muscle fatigue aiding diagnosis of muscle disease and for controlling prostheses.^7–9^ In 1993, Orizio coined the term MMG and reviews of the technique have since charted its development.^2,10–12^

The MMG technique is easier to use than EMG because it does not require pre-amplification, coupling gel, direct skin contact or such precise positioning. MMG therefore offers more practical, efficient, hygienic, reusable implementation for real-world (out of clinic) use. However, technical limitations due to interference of signals from artefacts have limited the progress of MMG research and clinical applications until recently. Novel signal processing techniques have been developed, which include hardware and software filtering strategies, alongside feature ranking/selection algorithms to remove mechanical artefacts and isolate muscle activity in the MMG signal.^12,13^

Following these technical advances in MMG hardware and software, their robustness in terms of validity and reliability needs to be examined. Standardised protocols can be followed for testing MMG signals against known measures of force and EMG during isometric and dynamic contractions to confirm known force/MMG/EMG relationships.^14–16^

Reliability of repeated testing on different days is also important to examine, so that the degree of error can be factored into determining true change over time or in response to an intervention. The present study aimed to examine the validity and reliability of MMG signals recorded using novel sensor and signal processing techniques.

## Methods

### Study design

The validation aspect of the study compared changes in MMG signals against EMG changes during different levels of force. The reliability aspect compared recordings made on two different days using a standardised protocol, in a test re-test reliability design.

### Participants

A sample of convenience of 18 healthy adults aged 27–82 years (mean 44.2, SD = 16.7) was studied (n = 7 males, n = 11 females).

Exclusion criteria were any musculoskeletal disorders or injuries, neurological or systemic conditions, skin disorders (e.g. psoriasis, allergies). Participants were recruited via various routes, including staff and students at the University, through a poster, from University of Southampton healthy adult participant database and through word of mouth via other participants.

Participants were provided with a participant information sheet and gave their written informed consent prior to being studied. The guidelines of the Declaration of Helsinki were followed and the rights, dignity, safety and well-being of participants were respected throughout the study. Ethical approval was obtained from the Faculty of Health Sciences Ethics Committee at the University of Southampton (Ethics No. 18039).

### Equipment

**Figure 1.**
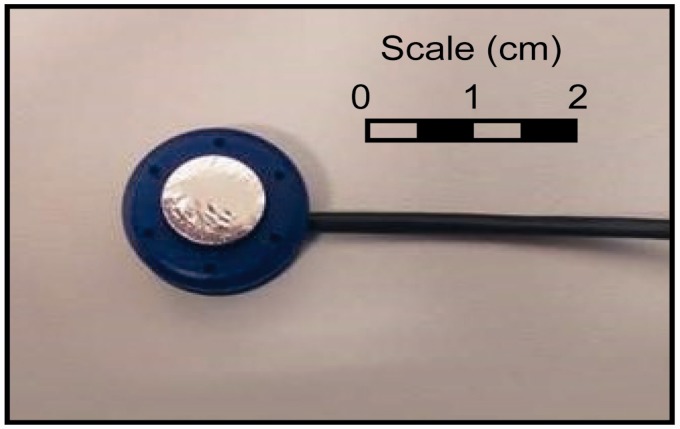
MMG hardware used in this study. The device is comprised of a clip/cap (blue) to compress all the parts together, a sleeve to keep the membrane (grey) taut, an acoustic chamber/housing and an electronic board which holds the microphone.

Three items of experimental equipment were used:

*MMG* – Muscle vibrations were measured using MMG sensors, each consisting of a microphone (Knowles SPU1410) and a conical chamber with a height of 5 mm and a diameter of 7 mm enclosed by a Mylar membrane (Figure 1). The MMG sensors employ a miniature silicon microphone (Knowles SPU1410LR5H-QB) consisting of an acoustic sensor, a low noise input buffer and an output amplifier.

*EMG* – Surface EMG (sEMG Biometrics SX230100) was used to compare with MMG, to examine known relationships with force and to compare the reliability of the two signals.

**Figure 2.**
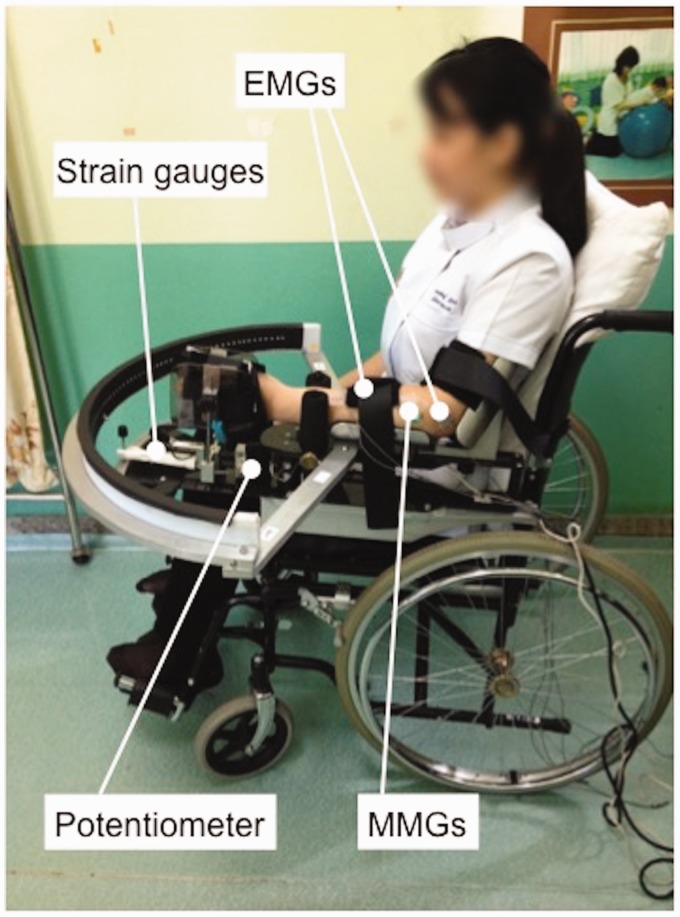
Participant set-up in the wrist rig showing force sensors, angle sensors, sEMG attached to the skin over forearm flexors and placement of MMG sensors over the forearm flexors. EMG: electromyography; MMG: mechanomyography.

*Wrist rig* – The wrist rig (Figure 2) is an instrumented neuromechanical measurement device consisting of an armrest attached to a chair with a potentiometer (angle sensor) and strain gauge (force sensor) and with two channels of sEMG. Wrist position is indicated by an LED pointer which allows the user to track a moving target (indicated by a blue LED) around a 120° horizontal arc. The wrist rig was developed to measure wrist motor impairments in stroke, including isometric flexor and extensor strength, motor control accuracy, wrist stiffness and muscle activation patterns during dynamic tracking tasks and response to rapid stretching (stretch reflex response) for spasticity. These indices were evaluated for test–re-test and inter-rater reliability and the sensitivity to distinguish between healthy individuals and stroke patients.^17^

### Technical developments to the wrist rig

**Figure 3.**
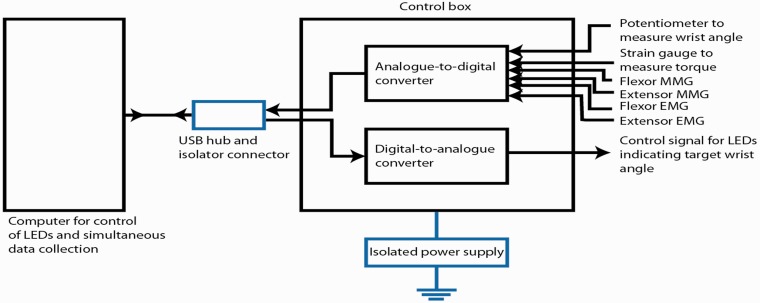
Schematic diagram of wrist wig equipment. EMG: electromyography; LED: light emitting diode; MMG: mechanomyography; USB: universal serial bus.

*Hardware –* Two MMG sensors were integrated with the wrist rig in order to compare EMG and MMG signals on wrist flexors and extensors (Figure 3). One analogue channel was used for each MMG sensor. The control unit of the wrist rig provided power supply to the MMG sensors at 3.3 V and measured their output voltage. The system underwent safety testing prior to the experiments.

*Software –* The software developed for the wrist rig was modified to acquire and process the signal from the MMG sensors. The graphical user interface was upgraded in order to display the MMG signal. In particular, the MMG signal was band-pass filtered and rectified for the purpose of visualisation.^18^

### Testing of the MMG component

All test procedures were carried out independently at the University of Southampton by one trained Research Physiotherapist (CM).

The validity of the MMG sensors was examined against known measures of isometric force (generated at the wrist) and EMG recordings of electrical muscle activity, following an established testing protocol for isometric muscle activity to confirm known force/MMG/EMG relationships.^15^

The neuromechanical rig (wrist rig) was used for isometric and dynamic testing.^17^ The participant was seated comfortably in the wheelchair, to which the wrist rig was attached. Following skin preparation using EMG SENIAM recommendations,^19^ surface EMG electrodes were placed over the flexor carpi radialis (FCR) on a line from the medial epicondyle of the elbow to the radial styloid process, one-third distal to the medial epicondyle.^17^ The extensor (extensor carpi radialis longus) EMG electrodes were placed on a line from the lateral epicondyle of the elbow to the second metacarpal, 5–7 cm distal to the lateral epicondyle.^17^ The MMG sensor was placed on the muscle belly close (distal) to the EMG electrodes. The muscle body was determined by clinical assessment by a chartered physiotherapist (CM).

The participant performed a pseudo random step-tracking task, which generated the data to examine the reliability of onset times. The task involved following a red light on the wrist rig, flexing and extending the wrist. The participant then performed an isometric task by flexing the wrist with maximal effort, pushing against a resistance for 3 s, during which force, and surface EMG and MMG were recorded over the FCR muscle. Three maximal contractions were performed and the highest value taken as the maximum. Percentages of maximal effort were calculated from the force signal and used as a target for submaximal contractions at 10, 25, 50 and 75% of maximum. Three contractions were performed at each level of effort (three contractions at five levels of effort, totalling 15 contractions). Rest periods (30–60 s, as required) were given between each set of contractions and (10–15 s) between each contraction. Each testing session lasted no longer than 90 min and decreased during the study, ranging from 45 to 90 min. Participants attended on two days, one week apart, on the same day of the week and at the same time of day, as far as possible.

### Signal processing

The EMG and MMG signals were pre-processed in the same way for consistency. First, the signals were decimated, second a 50 Hz notch filter was applied to the EMG, third a 10–50 Hz band pass filter was applied to the MMG, fourth an 80 Hz low-pass filter was applied to the torque, and finally the MMG signal was then rectified.^18^ The 50 Hz notch filter aims to remove AC interference from the power line for all measurements. The 10–50 Hz band pass filter applied to MMG measurements aims to remove the low-frequency bias and the high-frequency noise. These values have been selected as in Woodward et al.,^13^ since they represent the lower bound and the upper bound of the mean power frequency of MMG signals.^20^ The 80 Hz low-pass filter applied to the torque measurements aims to remove high frequency noise. This value was selected empirically and represents a good compromise between noise reduction and attenuation of high frequency signal components.

The onset time for MMG and EMG signals was calculated in the tracking task. Wrist extensor muscle onset timing was defined as the interval between the target light switching on (from a flexion position to an extension position) and the detected MMG/EMG onset, where the onset threshold was four standard deviations above a resting local baseline of extensor MMG/EMG. This was recorded for 1 s immediately prior to each extension target switching on during a step-tracking task. An algorithm (written in Matlab) was used for automated calculation of onset time and checking of all onset points was conducted by visual inspection.

The root-mean-square (RMS) values were calculated over 1 s for MMG and EMG signals during the isometric phases of the tasks.

### Data protection and anonymity

All data were anonymised and each participant was assigned an ID number, so that they could not be identified. Data were stored on a password-protected computer and only the research team had access to data. Data will be kept for 10 years after the study, following the policy of the University of Southampton.

### Data analysis

*Data management –* Data were entered into Excel files and summarised for the sample as means and standard deviations.

*Statistical analysis –* The data for the MMG and EMG signals were tested for normality of distribution using the Shapiro–Wilk test. The relationship between EMG and MMG signals was examined using correlation analysis (R^2^). Reliability was examined using the intraclass correlation coefficient (ICC) and Bland and Altman analysis. The ICC model used was a single measures one-way random effects where people effects are random.

## Results

### Reliability of MMG and EMG signal onset time between days

The delay in onset of MMG and EMG from the start of the tracking task (appearance of the target light) was reliable between the two days and similar between the two signals. The ICC for MMG was 0.78 and for EMG was 0.79. The 95% confidence intervals were MMG 0.519–0.913 and EMG 0.541–0.918.

**Figure 4.**
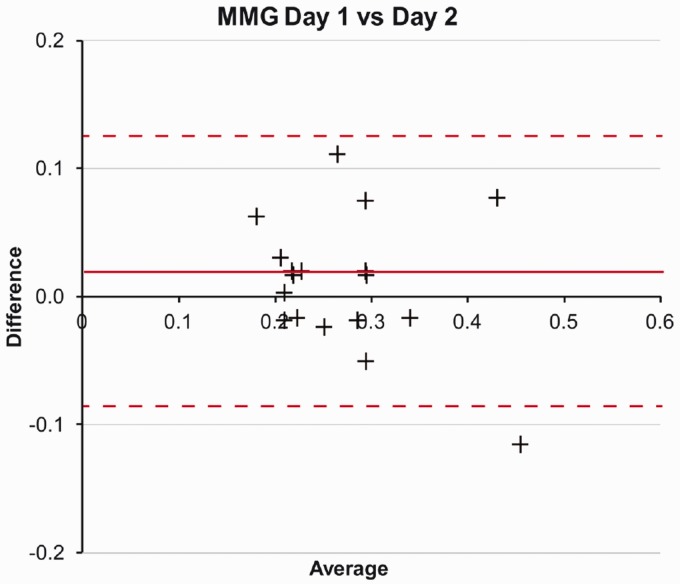
MMG Bland and Altman plot for difference between onset times (s) recorded on day 1 and day 2. MMG: mechanomyography.

**Figure 5.**
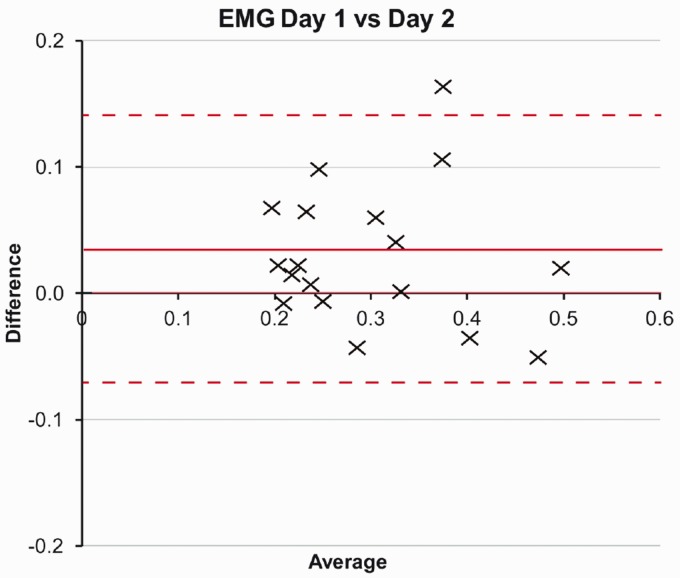
EMG Bland and Altman plot for difference between onset times (s) recorded on day 1 and day 2. EMG: electromyography.

Bland and Altman analysis plots did not reveal any systematic bias, as illustrated for MMG (Figure 4) and EMG (Figure 5). The mean difference for MMG was 0.0011 (limits of agreement 0.115 to −0.092) and EMG mean 0.0315 (0.141 to −0.079).

### Validity of MMG against force of contraction

**Figure 6.**
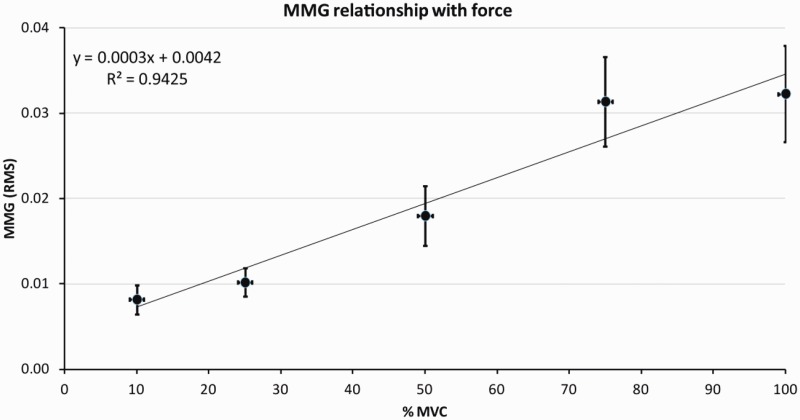
Relationship between force (% of maximal) and mechanomyography (RMS). Mean and standard error of the mean for values between 10 and 100% force. MMG: mechanomyography; MVC: Maximal Voluntary Contraction; RMS: root-mean-square.

The relationship between force of contraction and the MMG signal was highly correlated (R^2^=0.94), as illustrated in Figure 6.

## Discussion

This study examines the reliability and validity of recordings of mechanical muscle activity during upper-limb tasks using a newly designed and patent-filed MMG. The present findings demonstrate that MMG recordings made during voluntary contractions on different days are reliable and are also related to changes in force, indicating their validity for assessing mechanical muscle activity.

Evidence has previously reported MMG to have high reliability to measure muscle force contraction.^2,21^Previous literature has indicated that using MMG RMS has high between-day reliability (ICC=0.8) when compared with EMG to determine force (the more conventional means of recording surface muscle activity).^22^ However, additional evidence has been required to determine the between-day reliability of MMG with respect to measuring muscle onset timing. High between-day reliability of EMG to measure muscle onset timing has previously been demonstrated by Hodges and Bui,^23^ who reported that EMG had a high level of between-day reliability when utilising a visual inspection method to determine muscle onset timing. The present study demonstrated MMG signals were as reliable between days as EMG (MMG ICC = 0.78 and EMG ICC = 0.79) demonstrating our newly designed MMG is comparable with EMG to measure muscle onset timing.

Various factors affect reliability of recording of MMG signals, including contact pressure.^24–26^ muscle length/joint angle, temperature and positioning of the sensor. This is because greater MMG activity is recorded over the middle of the muscle belly although frequency is unaffected.^7^ These factors therefore need to be considered when using MMG sensors and standardised as much as possible: the level of precision necessary varying with the intended use. For example, laboratory investigations of muscle characteristics would require more precise recording conditions compared to biofeedback in field situations.

Placement of the MMG device over the muscle affects the nature of the signals recorded.^27,28^ It is therefore important to place the device consistently at the same site to ensure reliability of repeated recordings.

The strong relationship found between force of contraction and the MMG signal (R^2^=0.94) confirmed previous literature that MMG provides a valid indication of changes in force levels.^16,29^ The present study involved brief isometric contractions in previously rested muscle, which showed characteristic linear relationships between force and EMG, and force and MMG.^15,30^ However, when muscle is fatigued evidence suggests an alteration in the MMG and EMG signal parameters and % of MVC relationships.^10^ MMG provides a more accurate assessment of changes in force than EMG, due to dissociation that occurs between force and EMG when fatigue is present during isometric and dynamic contractions.^31,32^ This occurs due to higher neural effort being required to achieve a given force.

A limitation of the present study was that the motor tasks used for recording signals were not functional. The purpose was to standardise the recording conditions as far as possible. The wrist rig used for measuring muscle onset time of dynamic contractions restricted the plane of movement. The contractions used to generate different levels of force of contraction were isometric and also restricted in their direction. Reliability during more functional tasks needs to be examined, now that the performance of the sensor has been established for recording signals from muscle.

## Conclusions

Our MMG sensor produced reliable signals in terms of timing of muscle activity onset, comparable with the reliability of EMG signals, when a step-tracking task was repeated on different days. The MMG sensor signals were valid when compared with isometric force, confirming the MMG/force relationship documented in the literature. In the context of using the MMG sensors within the M-MARK project, the present study has demonstrated that our MMG sensor offers a valid and reliable measurement for measuring mechanical muscle activity for incorporation into a wearable device for stroke rehabilitation.
